# Low-Intensity Focused Ultrasound Alleviates Chronic Neuropathic Pain-Induced Allodynia by Inhibiting Neuroplasticity in the Anterior Cingulate Cortex

**DOI:** 10.1155/2022/6472475

**Published:** 2022-07-23

**Authors:** Bin Wang, Mo-Xian Chen, Shao-Chun Chen, Xiang-Jun Feng, Ye-Hui Liao, Yun-Xin Zhao, Jin-Shan Tie, Yao Liu, Li-Juan Ao

**Affiliations:** School of Rehabilitation, Kunming Medical University, Kunming, 650500 Yunnan Province, China

## Abstract

Low-intensity focused ultrasound (LIFU) is a potential noninvasive method to alleviate allodynia by modulating the central nervous system. However, the underlying analgesic mechanisms remain unexplored. Here, we assessed how LIFU at the anterior cingulate cortex (ACC) affects behavior response and central plasticity resulting from chronic constrictive injury (CCI). The safety of LIFU stimulation was assessed by hematoxylin and eosin (H&E) and Fluoro-Jade C (FJC) staining. A 21-day ultrasound exposure therapy was conducted from day 91 after CCI surgery in mice. We assessed the 50% mechanical withdrawal threshold (MWT_50_) using Von Frey filaments (VFFs). The expression levels of microtubule-associated protein 2 (MAP2), growth-associated protein 43 (GAP43), and tau were determined via western blotting (WB) and immunofluorescence (IF) staining to evaluate the central plasticity in ACC. The regions of ACC were activated effectively and safely by LIFU stimulation, which significantly increased the number of c-fos-positive cells (*P* < 0.05) with no bleeding, coagulative necrosis, and neuronal loss. Under chronic neuropathic pain- (CNP-) induced allodynia, MWT_50_ decreased significantly (*P* < 0.05), and overexpression of MAP2, GAP43, and tau was also observed. After 3 weeks of treatment, significant increases in MWT_50_ were found in the CCI+LIFU group compared with the CCI group (*P* < 0.05). WB and IF staining both demonstrated a significant reduction in the expression levels of MAP2, GAP43, and tau (*P* < 0.05). LIFU treatment on ACC can effectively attenuate CNP-evoked mechanical sensitivity to pain and reverse aberrant central plasticity.

## 1. Introduction

Neuropathic pain (NP) is caused by injury or disease of the somatosensory system [1]. Spontaneous pain, persistent or paralysis pain, induction pain, paresthesia, and pinprick sensation are clinical symptoms of NP. Approximately 6.9%–10% of patients worldwide suffer from chronic pain [2], which exerts a negative impact on their quality of life [3, 4]. Recent integrative neuroscience studies have found that chronic pain and acute pain operate through different central mechanisms, and central sensitization (CS) is the most important mechanism for chronic pain maintenance [5]. CS is an enhancement in the function of neurons and circuits in nociceptive pathways caused by the increases in membrane excitability and synaptic efficacy as well as by reduced inhibition. It is a manifestation of the remarkable plasticity of the somatosensory nervous system in response to activity, inflammation, and neural injury [6]. Pharmacotherapy for neuropathic pain is nonspecific and often insufficiently effective, and CS implies poor functioning of endogenous analgesia; thus, analgesic drugs are ineffective in controlling chronic pain with CS [6, 7]. In addition, drugs have many side effects after long-term use [8, 9]. Consequently, CS has revealed the role of the development of chronic pain in the central nervous system.

CS encompasses various related dysfunctions within the central nervous system, including altered sensory processing in the brain with a disrupted resting state functional connectivity in the default mode and salience networks and increased brain activity in areas known to be involved in acute pain sensations, including the anterior cingulate cortex (ACC). ACC is an important cortical area in sensory and cognitive research. In animal studies, cumulative evidence has revealed that the generation and maintenance of chronic pain and pain-related emotions are accompanied by long-term plastic changes within the ACC after peripheral injury [10–14]. It has been shown that central synaptic plasticity contributes to CS in chronic pain. In a spinal nerve ligation (SNL) rat pain model, maladaptive plasticity in the ACC brain region has been detected along with the progression of the pain response in rats. Furthermore, it has been found that the inhibition of the neural remodeling of the ACC brain region at both structural and functional levels by the CDK5/microtubule-associated protein 2 (MAP2) and CDK5/tau-NMDA2B pathways provides an analgesic effect [15]. In addition, many studies have reported that inhibiting synaptic plasticity or synaptic enhancement in the ACC region of mice can significantly alleviate the pain sensitivity response caused by peripheral nerve damage [16]. Consequently, plasticity of ACC strongly correlates with chronic neuropathic pain (CNP); it may be considered an important target for CNP drug intervention.

As a means of noninvasive neuromodulation, ultrasound can effectively regulate the activity of neurons in the central nervous system. In vitro experiments have shown that ultrasound stimulation activates neuronal activity and local potentials generated by neuronal charge. In animal experiments, ultrasound stimulation of the central nervous system has been used to treat diseases such as epilepsy, NP, Alzheimer's disease (AD), and Parkinson's disease (PD) by inhibiting the activity of neurons or charge and regulating synaptic plasticity [17–20]. In human trials, this neuromodulation technology has a direct effect on the primary somatosensory cortex, increasing brain activity in this area and improving sensory discrimination but also transiently and reversibly changing the activity of neurons in the subcortical and deep cortical areas [21, 22]. Therefore, the use of ultrasound for the treatment of related neurological disorders is becoming increasingly attractive.

Previous research has shown that low-intensity focused ultrasound (LIFU) has a significant analgesic effect on CNP mice due to central ACC regulation; however, the mechanisms underlying the alleviation of allodynia remain unknown. We used a CCI approach to create a mouse model of CNP and regulated the ACC region by LIFU to investigate the analgesic effect on allodynia and the probable underlying mechanism.

## 2. Materials and Methods

### 2.1. Laboratory Animals

Animal care and treatment protocols followed the *Guidelines for the Care and Use of Laboratory Animals* [23]. A total of 42 healthy male C57BL/6J mice (age, 8 weeks; weight, 20–25 g) were used in these experiments. To investigate the safety and effects of LIFU on neurons, we randomly allocated normal C57BL/6J mice to a LIFU(-) group and a LIFU(+) group (*n* = 6 mice/group). For the LIFU neuromodulation experiment, 30 healthy male mice were randomly divided into the following three groups: the sham, CCI, and CCI+LIFU groups (*n* = 10 mice/group). The mice were provided by the Department of Experimental Animals, Kunming Medical University. They were maintained in a standard animal room with a light/dark cycle of 12 h/12 h and were provided free access to food and water. The temperature of the animal room was 22 ± 2°C, and the relative humidity was 50%–70%. The experimental protocol was approved by the Experimental Animal Ethics Committee of Kunming Medical University (No. KMMU2021345).

### 2.2. Grouping and CNP Model

Prior to the study, the mice were adapted to the maintenance environment for 1 week. Thirty mice were then divided into the sham group, the model group, and the LIFU treatment group, with 10 mice in each group. Then, we used the CCI surgical method [24] to establish CNP models. Specifically, the sciatic nerve in the CCI group and the treatment group was ligated for 90 days. The mice were anesthetized with isoflurane (Sigma-Aldrich, St. Louis, Missouri, USA) and laid on a heating pad. In the prone position, the surgical site was prepared by shaving the posterolateral side of the right hind limb, and then, three applications of 75% ethanol were applied to the site. An incision (1 cm) was then made proximal to the right hind limb. The nerve was then uncovered using a splitting approach on the bicep femoris, and three ligatures (gut ligatures 6.0, Jinhuang, Shanghai, China) were tied at 1 mm long intervals. The deep and superficial muscles were reapproximated by applying an interrupted stitching technique using 4.0 chromic gut (Stoelting Co, Wood Dale, IL). The skin was closed using 4.0 silk sutures (Ethicon, Somerville, NJ). For the sham group, the right sciatic nerve was exposed using the same methods, but the nerve was not ligated. After surgery, the mice were returned to their original cages to continue feeding.

### 2.3. Ultrasound Treatment Protocol

**Figure 1:**
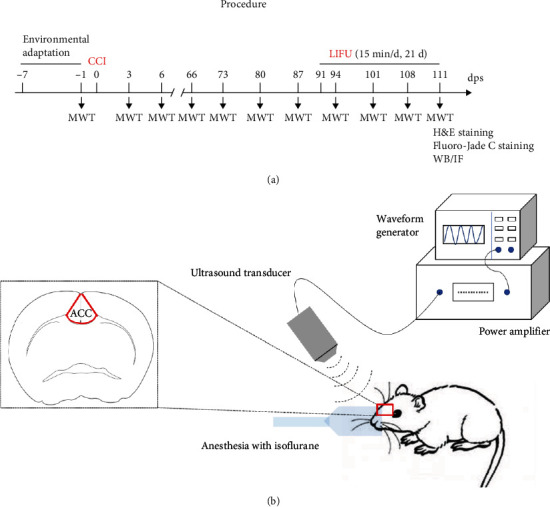
(a) Procedure used for our experiments. Dps: days postsurgery. (b) Schematic diagram of low-intensity focused ultrasound (LIFU) stimulation.

The mice commenced LIFU stimulation on day 91 after CCI modeling. The treatment group underwent LIFU stimulation under anesthesia with isoflurane via a nose cone. We used an electric shaver and depilatory cream to remove the hair on the skin of the head without damaging the integrity of the skin or skull. We calculated the focal length to prepare the collimator of the LIFU transducer, which was placed directly in the area of the ACC (the Bregma point). A waveform signal was generated by a waveform generator (DG4202, RIGOL, China) and was amplified with a 50 W power amplifier (Dahan Radio Studio, China). The amplified signal then activated the ultrasound transducer (Figure 1(b)). An ultrasonic coupling agent (Aquasonic; Parker Laboratories, Fairfield, NJ, USA) was filled between the ultrasound transducer and the skin of the head to evacuate air bubbles. For LIFU stimulation, we used a focused transducer (4 MHz), with an acoustic intensity of 0.95 MPa, a duty cycle (DC) of 10%, and a pulse repetition frequency (PRF) of 1.5 kHz [25]. LIFU stimulation was administered for 15 min/day for 21 days (Figure 1(a)). We measured the acoustic intensity of the beam by using a hydrophone (HNR 0500; Onda, Sunnyvale, CA, USA). Mice in the model group received the same LIFU stimulation as those in the treatment group under anesthesia; however, LIFU was turned off during the treatment. Animals in the sham group received isoflurane anesthesia for 15 min and were then returned to their original cages.

### 2.4. Evaluation of Mechanical Withdrawal Threshold

The mice were placed in a 5 × 5 × 8 cm^3^ plexiglass grid and were allowed to adapt to the testing environment for 10 min prior to experimentation. In accordance with methods described previously [26], we used the “up-down” method to evaluate the 50% mechanical withdrawal threshold (MWT_50_) of mechanical allodynia in mice. Von Frey filaments (VFFs) (Stoelting, Wood Dale, IL, USA) with ascending degrees of stiffness (0.02, 0.04, 0.07, 0.16, 0.4, 0.6, 1.0, 1.4, and 2 g) were used to stimulate the bottom of the right paw in each mouse. The stimulus intensity started at 0.6 g, and an appropriate external force was applied to make the VFFs bend 90°; this was maintained for 5 s. A positive reaction was defined as cases wherein a test mouse was seen to lick, raise, or retract its paws. Then, we selected larger or smaller VFFs for stimulation based on the response of each mouse. When a reaction different from the previously described one was observed, we repeated the VFFs four more times to end the MWT_50_ test. If the force was >2.0 g or <0.02 g, it was recorded as 2.0 g or 0.02 g, respectively. Then, we calculated the pain threshold as follows:

(1) $$\begin{array}{c} {\text{MWT}}_{50}=\left.\frac{\left(10^{\left[xf+k\delta \right]}\right)}{10000}\right), \end{array}$$

where *xf*, *k*, and *δ* represent the mean intensity, stimulus coefficient, and log value of the adjacent stimulus, respectively, and *xf* and *K* can be obtained from statistical tables; in this study, *δ* was 0.24.

### 2.5. Histological Analysis

The mice were anesthetized with an intraperitoneal injection of 1% pentobarbital sodium, followed by cardiac perfusion with 0.9% normal saline and 4% paraformaldehyde. Fresh brain tissues were fixed in a fixative solution for 48 h and then dehydrated in various concentrations of absolute ethanol (75%, 85%, 90%, 95%, and 100%) and different concentrations of xylene. Wax-soaked brain tissues were then embedded in an embedding cassette, and tissue sections with a thickness of 5 *μ*m were cut on a paraffin slicer. For hematoxylin and eosin (H&E) staining, the sections were first dewaxed in xylene I (10 min) and xylene II (10 min), followed by absolute ethanol I, anhydrous ethanol II, 95% alcohol, 90% alcohol, 80% alcohol, and 70% alcohol for 5 min each. The sections were then rinsed in double-distilled water for 5 min and stained in hematoxylin for 5 min. Next, they were rinsed in double-distilled water, differentiated in 1% hydrochloric acid alcohol for a few seconds, rewashed in double-distilled water, incubated in 0.6% ammonia water (back to blue), and then rinsed in double-distilled water before staining in eosin for 3 min. Finally, the sections were washed with 95% alcohol I, 95% alcohol II, absolute ethanol I, absolute ethanol II, xylene I, and xylene II for 5 min each time. The slides were sealed with neutral gum sealing tablets, and pathological changes in the sections were investigated under a light microscope (Olympus Corporation, Tokyo, Japan).

For Fluoro-Jade C (FJC) staining, entire mouse brains were acquired as described earlier. However, the brains were fixed in 4% paraformaldehyde for 48 h and dehydrated for 24 h in various concentrations of sucrose solution (10%, 20%, and 30%) before being embedded at optimal cutting temperature (OCT). Then, the frozen tissues were cut into sections with a thickness of 10 *μ*m using a frozen microtome. For FJC staining, we used a commercial kit (Biosensis, Adelaide, Australia), and the sectioned tissues were immersed in a mixture of solution A and 80% ethanol for 10 min. This was then incubated in a 70% ethanol solution for 2 min and then in a mixture of solution B and double-distilled water for 10 min. Next, the mixture was incubated with solution C and double-distilled water for 10 min, washed with double-distilled water three times (1 min each time), and dried on a heater at 50°C–60°C for 5 min. Finally, the sections were cleared by xylene treatment for 2 min. DPX mounting medium was added dropwise, and the sections were covered with cover glass. Then, we observed the sections via fluorescence microscopy (Olympus Corporation, Tokyo, Japan).

### 2.6. Western Blotting (WB)

Following the last behavioral test, the mice were anesthetized with an intraperitoneal injection of excessive sodium pentobarbital, followed by cardiac perfusion with 0.9% normal saline. The ACC brain tissues were then dissected, lysed with radioimmunoprecipitation assay (RIPA) buffer on ice for 30 min, and homogenized with an ultrasonic cell crusher. The lysate was then centrifuged at 12000 rpm at 4°C for 30 min. The supernatant was taken, and the total protein concentration was determined with a bicinchoninic acid (BCA) assay kit (Biomed, Beijing, China). Sodium dodecyl sulfate polyacrylamide gel electrophoresis (SDS-PAGE) was then used to separate proteins, which were then transferred to polyvinylidene difluoride (PVDF) membranes (Millipore-Sigma, Burlington, MA, USA). The membranes were then blocked with 5% skimmed milk and incubated overnight at 4°C with primary antibodies against MAP2 (1 : 1000; Cell Signaling Technology (CST), Danvers, MA, USA), GAP43 (1 : 1000; CST), tau (1 : 1000; Proteintech, USA), and *β*-tubulin (1 : 2000; Abcam, Cambridge, UK). The next day, the membranes were washed three times with tris-buffered saline with 0.1% Tween® 20 (TBST) (15 min per wash) and then incubated with horseradish peroxidase- (HRP-) labeled goat anti-rabbit/anti-mouse IgG (1 : 5000) HRP-linked antibody (1 : 2000; CST) at room temperature (RT) for 2 h. Then, the membranes were washed three times with TBST (15 min per wash). Finally, the chemiluminescence (ECL; Tanon, Shanghai, China) imaging method was used to reveal protein bands, and ImageJ (US National Institutes of Health (NIH), Bethesda, MD, USA) was used to analyze the gray level of each protein band.

### 2.7. Immunofluorescence (IF)

The mice were anesthetized with an overdose of sodium pentobarbital intraperitoneally; they were then perfused (via the heart) with 0.9% normal saline and fixed with 4% paraformaldehyde. The brains were then removed, fixed for 48 h in 4% paraformaldehyde, and dehydrated for 24 h with a gradient of 10%, 20%, and 30% sucrose concentrations. The tissues were then embedded at OCT, and 10 *μ*m thick frozen sections were cut with a microtome. For analysis, the sections were warmed to RT, washed in phosphate-buffered saline (PBS) for 10 min, blocked with 10% goat serum for 2 h, and then incubated at 4°C overnight with primary antibodies against c-fos (1 : 300; Proteintech, USA), MAP2 (1 : 200; CST), GAP43 (1 : 200; CST), tau (1 : 200; CST), and NeuN (1 : 500; Abcam). The next day, the sections were warmed to RT, washed with PBST for 15 min, and then incubated with secondary antibody (anti-rabbit IgG (heavy+light (H+L) chain), F [ab′]2 fragment (Alexa Fluor 488 conjugate); anti-mouse IgG (H+L chain), F [ab′]2 fragment (Alexa Fluor 594 conjugate)) at RT and in the dark for 1.5 h. Next, the sections were washed with PBST for 15 min and incubated with 4′,6-diamidino-2-phenylindole (DAPI; Sigma, USA) for 20 min. Finally, we observed the sections and acquired photographs with a fluorescence microscope (Olympus Corp, Tokyo, Japan). ImageJ software (US National Institutes of Health (NIH), Bethesda, MD, USA) was used to analyze the optical density of the positive area.

### 2.8. Statistical Analysis

Statistical analysis was performed using SPSS 25.0 software (IBM Corp, Armonk, NY, USA). GraphPad Prism software version 8.0 (GraphPad Software, Inc., San Diego, CA, USA) was used to generate graphs. The raw data obtained were all expressed as means ± standard error of mean (SME). WB bands and IF were analyzed using one-way analysis of variance (ANOVA) with Tukey's post hoc test. Comparisons of two groups were performed by a two-tailed unpaired *t*-test, while behavioral data were analyzed by two-way repeated-measures ANOVA, followed by Bonferroni's test for post hoc comparisons. Two-tailed *P* values < 0.05 were considered statistically significant.

## 3. Results

### 3.1. LIFU Stimulates the ACC in Each Group of Mice in a Safe Manner

**Figure 2:**
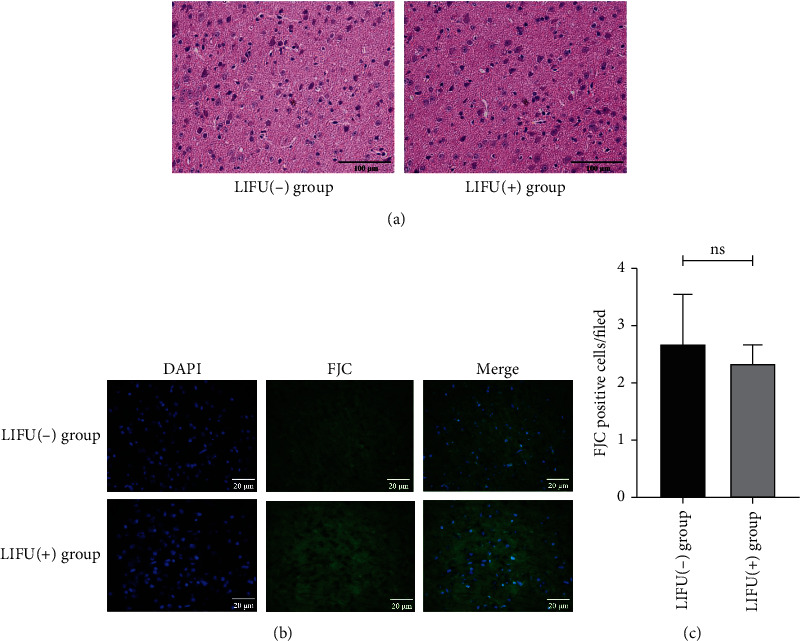
Safety evaluation of LIFU stimulation (×400). Scale bars, 100 *μ*m and 20 *μ*m. (a) Hematoxylin and eosin (H&E) staining showed no evidence of edema, hemorrhage, or cell necrosis; *n* = 3 per group. (b, c) Fluoro-Jade C (FJC) staining showed that there was no significant difference in the number of FJC-positive cells between the LIFU(-) and LIFU(+) groups. Each symbol represents the mean ± SEM; independent-sample *t*-tests; *n* = 3 mice per assay.

H&E staining of the ACC brain tissues (Figure 2(a), ×400) in the LIFU(-) and LIFU(+) groups showed that there was no definitive bleeding, nerve cell swelling, pyknosis, coagulative necrosis, or emptying. We also stained brain tissues with FJC, a marker of neuronal degeneration. There were no significant differences in the number of FJC-positive cells after stimulation between the LIFU(-) and LIFU(+) groups (Figures 2(b) and 2(c)). This suggests that LIFU stimulates the ACC brain area in a safe manner.

### 3.2. LIFU Significantly Activates Neurons in the ACC Region

**Figure 3:**
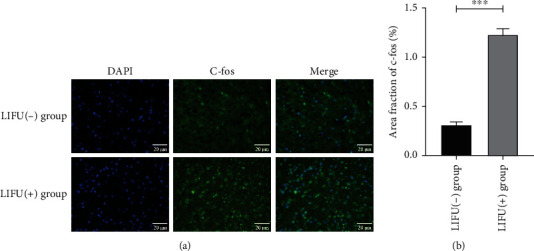
Expression of c-fos (a, b) in the anterior cingulate cortex (ACC) after low-intensity focused ultrasound (LIFU) stimulation. LIFU activated neurons in the ACC (immunofluorescence (IF), ×400). Scale bar, 20 *μ*m. ^∗∗∗^*P* < 0.0001. Each symbol represents the mean ± SEM; unpaired *t-*tests; *n* = 3 rats per assay.

To determine the effects of LIFU on neurons, c-fos expression was examined in the stimulation region. Compared with the LIFU(-) group, we found an increased fluorescence of c-fos in most neurons after LIFU stimulation (*P* < 0.05) (Figures 3(a) and 3(b)), indicating that LIFU significantly activated neurons.

### 3.3. LIFU Significantly Alleviates Allodynia in CNP Mice

**Figure 4:**
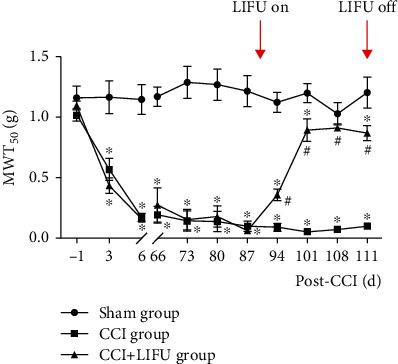
Effects of LIFU stimulation on the ACC in chronic neuropathic pain (CNP) model mice. 50% mechanical withdrawal threshold (MWT_50_) significantly decreased in the CCI and CCI+LIFU groups after CCI surgery when compared with the sham group until the end of the study. After 21 days of LIFU treatment, the MWT_50_ increased when compared with the CCI group. Each symbol represents the mean ± SEM; ^#^*P* < 0.05 compared with the CCI and CCI+LIFU groups; ^∗^*P* < 0.05 compared with the sham group. Two-way repeated-measures ANOVA, followed by the Bonferroni test; *n* = 10 per group.

Compared with the sham group, the MWT_50_s in the CCI and CCI+LIFU groups decreased after surgery on the third day and dropped to the lowest levels on the sixth day; the MWT_50_s values were 0.18 ± 0.03 g and 0.16 ± 0.03 g, respectively (*P* < 0.05). The reduction of MWT_50_ in the CCI group was sustained until the end of the LIFU stimulation period. Following LIFU stimulation, the MWT_50_ gradually increased, eventually becoming significantly higher in the CCI+LIFU group (0.90 ± 0.10 g) than in the CCI group (0.06 ± 0.01 g) after 10 days of LIFU stimulation (*P* < 0.05); the MWT_50_ then remained stable until the end of LIFU stimulation. However, the MWT_50_ in the CCI+LIFU group was significantly still lower than that in the sham group (*P* < 0.05; Figure 4). These results show that LIFU can alleviate mechanical hyperalgesia caused by CCI in CNP mice.

### 3.4. LIFU Stimulation Significantly Reduces the Expression of MAP2, GAP43, and Tau Proteins in the ACC

**Figure 5:**
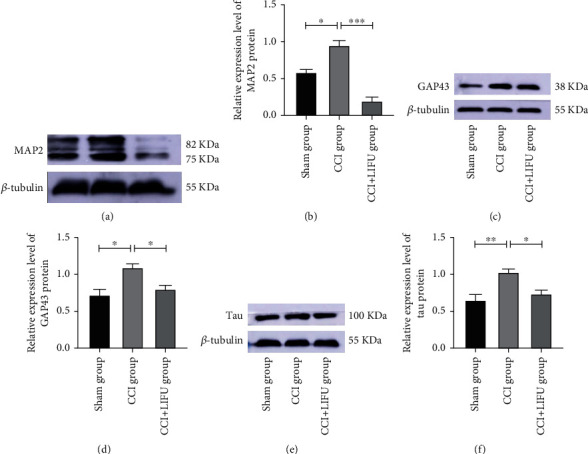
Western blotting (WB) analysis of microtubule-associated protein 2 (MAP2) (a, b), growth-associated protein 43 (GAP43) (c, d), and tau (e, f) expression in the ACC in different groups after 21 days post-LIFU treatment. Values were normalized to *β*-tubulin. Each symbol represents the mean ± SEM; ^∗^*P* < 0.05, ^∗∗^*P* < 0.01, and^∗∗∗^*P* < 0.001. One-way ANOVA; *n* = 5 rats per assay.

**Figure 6:**
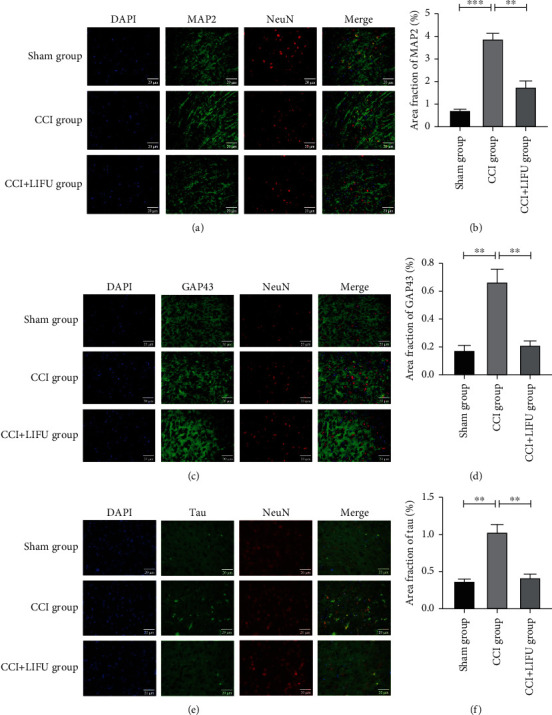
Expression of MAP2 (a), GAP43 (c), and tau (e) in the ACC of mice in different groups (immunofluorescence (IF), ×400). Scale bar, 20 *μ*m. The expression of MAP2 (b), GAP43 (d), and tau (f) in the ACC of mice in different groups after 21 days of LIFU treatment, as detected by IF. Each symbol represents the mean ± SEM; ^∗^*P* < 0.05,  ^∗∗^*P* < 0.01, and^∗∗∗^*P* < 0.001. One-way ANOVA; *n* = 5 rats per assay.

CS is an important mechanism of chronic pain that manifests as neuroplasticity. Neuroplasticity-related proteins include MAP2, GAP43, and tau. In this study, WB showed that the expression levels of MAP2, GAP43, and tau increased significantly in the CCI group (*P* < 0.05) (Figures 5(a)–5(f)). The expression levels of MAP2, GAP43, and tau decreased significantly after 21 days of LIFU treatment when compared to the CCI group (*P* < 0.05; Figures 5(b), 5(d), and 5(f)). IF also showed that the expression levels of MAP2, GAP43, and tau increased significantly in the CCI group (*P* < 0.05) (Figures 6(a)–6(f)). MAP2, GAP43, and tau levels decreased significantly after 21 days of LIFU stimulation when compared to the CCI group (*P* < 0.05; Figures 6(b), 6(d), and 6(f)).

## 4. Discussion

In this study, we used CCI to create a CNP mouse model as this is a simple technique that is easy to replicate. The CNP model developed by CCI is comprehensive, and the mechanical and heat pain sensitivity thresholds of the afflicted limbs of the animals are significantly lower than that in the sham group after surgery [27]. The long-term experimental protocol of 21 days of LIFU treatment from day 91 in the current study was based on the results from a previous report of our team [25]. In Feng et al.'s study, in the short-term experiment (21 days of LIFU treatment on ACC from day 6 after CCI injury), the focused ultrasound- (FUS-) induced mechanical analgesic effects appeared at 2 weeks following the surgery. However, an earlier appearance of FUS effects in the long-term experiment (21 days of LIFU treatment from day 91 after CCI) was observed on day 94 after CCI. In a rodent NP model, CS may be indicated by pain-induced generation and maturation of the potentiation of synaptic responses in the ACC and the development of allodynia at 1–4 weeks following nerve injury, as well as by the development of anxiodepressive-like behaviors at 5–8 weeks after injury [28]. This may explain the difference in the time of onset of FUS effects between the short- and long-term experiments and indicate that the ACC or CS may be the optimal target for pain improvement in the long-term experiments rather than in the short-term experimental design.

Therapeutic ultrasound has been widely used in clinical and scientific research due to its unique biological advantages and satisfactory efficacy in the treatment process [29, 30]. FUS is a noninvasive targeting therapy that uses a novel concave head to concentrate ultrasound energy into a range of millimeter diameters [31]. Unlike the thermal effects of high-intensity focused ultrasound (HIFU) [32], the biological effects of LIFU with regard to neuromodulation are primarily mechanical and can activate or inhibit neuronal activity by altering the state of mechanically sensitive ion channels embedded in cell membranes [33, 34]. Over the past decade, ultrasound stimulation of neurons has shown numerous advantages over electrical stimulation. In order to observe the changes in the functional state of neurons, most studies have detected the expression of c-fos in neurons as a marker of neuronal activity [35]. Qi et al. [36] selected low-intensity ultrasound to stimulate auditory neurons in vitro; they found that irrespective of whether low-frequency or high-frequency ultrasound was used, action potentials were produced in the cultured neurons. Furthermore, the expression levels of c-fos protein increased, as indicated by fluorescence staining, and neurons were significantly activated, as indicated by ultrasound. In addition, we showed that LIFU stimulation on ACC of normal mice increased the number of c-fos-positive cells in this region compared with non-LIFU controls by IF staining (Figure 3). The limitation is that we only examined the changes between LIFU and sham stimulation in normal mice but not in the CCI model. A previous study conducted by our team also showed that the numbers of c-fos-positive cells and GAD65-positive cells (a marker of synaptic activity) increased, respectively, after 0.5 MPa and 1.5 MPa LIFU stimulation compared with that after stimulation with 0 MPa (negative stimulation) in the lumbar region of the spinal cord, producing a similar result that LIFU activates neurons significantly. These findings demonstrate that LIFU may activate the neuronal cells and GABAergic terminals in the brain or spinal cord. In addition, LIFU can be used as a new alternative treatment strategy; its safety profile and practicality are very attractive [37]. Liao et al. [38] investigated spinal cord tissues from both normal rats and a rat model of pain and found that LIFU did not cause edema, bleeding, or the activation of glial cells. There is also evidence that LIFU stimulation does not cause significant damage to the cortex or hippocampus, immune cell infiltration, or changes in the number and morphology of glial cells [39]. In the present study, we investigated the safety of LIFU stimulation in the target brain area and found that there was no obvious swelling or apoptosis in the neuronal cells, as demonstrated by H&E and FJC staining. This also confirmed the safety of LIFU in the neuromodulation process and showed that this technique can safely stimulate the ACC area.

Central mechanisms are attracting a lot of attention as research progresses because they play an important role in preventing and developing pain. The ACC, located at the front of the corpus callosum, is involved in the limbic system and the prefrontal cortex (PFC) [40]. This area of the brain is not only associated with the formation of memories, emotional responses, motor control, and cognitive behavior but also involved in encoding nociceptor receptor stimulation, as seen in several rodent models of chronic pain in which chemical or electrolytic stimulation of the ACC effectively attenuated pain-related behavioral responses [40, 41]. Moon et al. [42] implanted an optical cable in the ACC region of rats with trigeminal neuralgia by using an adenovirus and found that the mechanical pain sensitivity threshold and cold pain sensitivity threshold scores of the rats increased after light stimulation; in addition, there was obvious pain relief, which confirmed the important role of ACC in analgesia. Recently, Feng et al. [25] discovered that LIFU regulated the ACC, as it was possible to effectively reduce the effect of MWT_50_ in the long-term experiment, but there were no significant changes in the thermal withdrawal threshold of the bilateral sides of the CNP model induced by CCI after 21 days of LIFU treatment on ACC. These findings suggest that the ACC brain region plays an important role in CNP, and its regulation may be a viable method to alleviate CNP allodynia. In our study, mechanical pain sensitivity symptoms were observed after surgery in CNP mice and were maintained until treatment was completed. The mechanical pain sensitivity of CNP mice improved significantly after LIFU treatment, thereby confirming the analgesic effect of LIFU on CNP-induced allodynia.

More detailed cell and molecular experiments with rodent models have revealed changes in neuronal and synapse remodeling in many areas of the brain involved in pain management, including the dorsal horns of the spinal cord and the cortical structures [43–45]. These advanced centers of the brain focus broadly on neural and synaptic remodeling of the ACC. At the synaptic level, potentiation of excitatory transmission caused by injuries may be mediated by the enhancement of glutamate release from presynaptic terminals and potentiated postsynaptic responses of AMPA receptors [9]. After long-term research, Lu et al. [46, 47] have found that chronic pain can cause changes in synaptic remodeling in the cerebral cortex and is closely related to pain perception and anxiety behavior, while the long-term potentiation (LTP) of synaptic transmission in the ACC region of the cerebral cortex is an important molecular mechanism for chronic pain. Furthermore, their team has recently discovered that increasing oxytocin content in ACC can selectively reduce chronic pain and eliminate pre-LTP that causes anxious behaviors and anxiety-related behaviors in mice with chronic pain associated with common peroneal nerve (CPN) ligation, confirming the effect of improving chronic pain by modulating central nervous system remodeling [48]. Um et al. [49] found that optical imaging techniques revealed a considerable increase in the neuronal response to peripheral stimulation in the ACC brain region of a rat model of CCI-induced chronic pain; when rapamycin inhibitors were injected into this brain area, the mechanical pain sensitivity response was significantly improved, confirming that the mechanism for relieving chronic pain may be to suppress synaptic plasticity induced by neuropathic pain by downregulating the mTOR signaling pathway, which could be a new strategy for treating chronic pain. Wang et al. [50] confirmed that the number of functional glutamatergic synapses increased along with new neural circuit formation and strengthening of the projection from the medial thalamus to the ACC. Structural remodeling was detected in the ACC, manifested by an increase in dendritic spine density and the number of synapses in a rat model of CCI-induced pain, thereby mediating the occurrence of pain. Furthermore, recent research has found that the enhancement of neural remodeling in the ACC caused by injecting trinitrobenzenesulfonic acid into the pancreatic duct is involved in the development and maintenance of visceral pain sensitivity and anxiety in rats, which supports a strong link between ACC neuroplasticity and chronic pain [51]. In our present study, WB and IF staining both demonstrated that the expression levels of neuroplasticity-related proteins (MAP2, GAP43, and tau) decreased significantly. This finding suggests that LIFU treatment on ACC can effectively attenuate CNP-evoked mechanical sensitivity to pain and reverse the aberrant central plasticity. The results of this study provide further research directions for the treatment of CNP by LIFU.

Overall, we demonstrated that (1) LIFU can be used to safely stimulate the ACC, (2) LIFU significantly improves mechanical pain sensitivity symptoms of the affected side in mouse models of CNP by stimulating the ACC region, (3) LIFU affects the expression levels of MAP2, GAP43, and tau proteins in the ACC area, and (4) the mechanism by which LIFU alleviates the CNP-induced allodynia may be achieved by reversing aberrant central remodeling.

## 5. Limitations

There are some limitations that need to be considered. First, we only measured MWT_50_, but the thermal allodynia thresholds and emotional and cognitive behaviors were not tested in mice. Second, this study only examined the morphology of neural remodeling but did not detect changes in neuronal function. The long-term effects of LIFU on neural cells were not studied further. Finally, we found that LIFU can influence the expression levels of MAP2, GAP43, and tau; however, we did not investigate the molecular mechanisms by which LIFU influences neuroplasticity. Thus, further experiments are needed.

## 6. Conclusions

We found that LIFU can be used safely to stimulate the ACC and alleviate CNP-induced allodynia. Moreover, LIFU analgesia may be related to the suppression of neuroplasticity.

## Data Availability

The data used to support the findings of this study are available from the corresponding authors upon request.

## Abbreviations

ACC: Anterior cingulate cortex
AD: Alzheimer's disease
CNP: Chronic neuropathic pain
CS: Central sensitization
CCI: Chronic constrictive injury
CPN: Common peroneal nerve
DC: Duty cycle
FJC: Fluoro-Jade C
GAP43: Growth-associated protein 43
HIFU: High-intensity focused ultrasound
LIFU: Low-intensity focused ultrasound
MWT_50_: 50% mechanical withdrawal threshold
MAP2: Microtubule-associated protein 2
NP: Neuropathic pain
PD: Parkinson's disease
PFC: Prefrontal cortex
SNL: Spinal nerve ligation
VFFs: Von Frey filaments.
